# Use of Anticoagulants in Patients With Non-Valvular Atrial Fibrillation Who Are at Risk of Falls

**DOI:** 10.7759/cureus.10336

**Published:** 2020-09-09

**Authors:** Layla Shanah, Sohaip Kabashneh, Samer Alkassis, Hammad Ali, Tanveer Mir

**Affiliations:** 1 Internal Medicine, Wayne State University/Detroit Medical Center, Detroit, USA

**Keywords:** non valvular atrial fibrillation, anticoagulation, falls, stroke, intracranial hemorrhage

## Abstract

Atrial fibrillation (AF) is a relatively common clinical entity with significant morbidity and mortality, particularly in the elderly. Stroke is one of the most significant complications of AF, which can be prevented with the use of anticoagulation. Elder population are at an increased risk of falls and the use of anticoagulation in this group can lead to intracranial hemorrhage. Therefore, it is unclear whether patients at high risk of falls should be anticoagulated. This review article discusses the epidemiology of AF and falls in the elder population, and whether the benefit of anticoagulation outweighs the risks in this group.

## Introduction and background

Atrial fibrillation (AF) is a cardiac arrhythmia defined by the presence of disorganized atrial activity with an irregularly irregular ventricular response on electrocardiogram. It is the most frequently encountered arrhythmia affecting 33.5 million individuals worldwide, and the risk increases in the elder population [1,2]. AF is associated with an increased risk for adverse events, including stroke, as well as heart failure and dementia [3]. The management of AF typically includes rate or rhythm control in addition to anticoagulation in selected patients.

Falls are common in the elderly, both in the community and inpatient settings, and can also lead to diminished quality of life. It has been found that in adults 65 years and older in a community setting, one-third fall at least one time per year [4].

Though the elderly are at higher risk for both the development of atrial fibrillation and subsequent stroke, many elderly patients are not prescribed anticoagulation for fear of bleeding complications secondary to falls. For many physicians, the potential risks of anticoagulation seem to outweigh the benefits and as such there may be unnecessary precautions taken when choosing not to use anticoagulation in elderly patients. The purpose of this review is to highlight the literature on the management of anticoagulation in the elderly population with atrial fibrillation, who are at the highest risk for strokes, but may be prone to falling, placing them at a potential increased bleeding risk.

## Review

Prevalence and burden of atrial fibrillation

AF is one of the most frequently encountered arrythmias. The prevalence of AF increases with increasing age. It is anticipated that in the United States (US), as the population from the post-World War II era ages, the number of individuals with AF will increase by 2.5-fold with 5.6 million adults having been diagnosed with AF by the year 2050, three million of these patients will be 80 years of age or older [1]. 

AF affects many elderly patients with sometimes devastating sequela. In order to assess the extent of disease burden affiliated with AF worldwide, Chugh et al. performed a systematic review of global epidemiological data on AF that approximated 20.9 million men and 12.6 million women to be living with AF in 2010 [2]. Furthermore, the study was able to differentiate differences in prevalence based on age with men between the ages of 75-79 having a twofold higher prevalence than those 65 to 69 years of age. When a comparison was made to men 55 to 59 years old, those in the eldest group had a prevalence that was fivefold higher [2]. A large cross-sectional study of adults 20 years of age and older in California by Go et al. also found the prevalence of AF to have a solid correlation with age with the prevalence of AF increasing with age [1]. AF was estimated to be diagnosed in approximately 4% of people 60 years and older and 9% of people 80 years and older. In addition, the study projected that the burden of disease will only continue to increase in the coming years as the current population ages [1]. Feinberg et al. approximated the prevalence of AF based on age from population-based surveys in the United States in conjunction with US census data [5]. The prevalence of AF was 2.3% in people older than 40 years and 5.9% in those older than 65 years. Importantly, the study showed that 70% of those affected by AF were between the ages of 65 and 85 [5]. From the studies mentioned and similar studies, it is clear that the prevalence of AF increases with age and as the number of elderly in the US population increases so too will the disease burden from AF. 

Atrial fibrillation is a strong risk factor for stroke particularly in the elderly

Stroke is one of the most detrimental and frightening complications of AF. Wolf et al. studied the incidence of stroke in 5,070 participants in the Framingham Study [6]. They found that when a comparison was made between patients without AF, those individuals with AF had a nearly fivefold increase in the incidence of stroke. In addition, the presence of AF in those individuals with underlying coronary artery disease or heart failure increased the risk of stroke by twofold in men and threefold in women. In fact, AF was the only cardiovascular disorder to independently influence stroke incidence in individuals aged 80 to 89 years old. Moreover, the attributable risk of stroke from AF increased from 1.5% for persons between the ages of 50-59 years to 23.5% for persons between the ages of 80-89 years. It was concluded from this study that the elderly with AF are especially susceptible to stroke [6]. Bjorck et al. investigated the attributable risk of stroke in patients with AF in the Swedish region [16]. They found that the attributable risk increases with age from 4.6% in those between the ages of 50 to 59 years to more than 20% in those 80 to 89 years [2,3].

There are clinical classification tools that have been developed to assess the risk of stroke in patients with AF, perhaps the most popular being the CHADS2 and CHA2DS2-VASc scores, which give an approximation of the annual stroke risk. The purpose of these scores is to provide a recommendation for decision making regarding anticoagulation therapy [7,8]. Despite these recommendations, many physicians choose not to anticoagulate older patients for fear of bleeding risk secondary to falls.

Patients with atrial fibrillation have worse outcomes from stroke

Lin et al. examined the stroke severity in patients with AF from the original Framingham cohort, focusing both on mortality and disability in stroke patients [9]. It was found that patients with AF who had strokes had a 25% 30-day mortality compared to non-AF patients at 14%. Those patients with AF were not only found to have poorer survival, but also an increase in recurrences during the year following the initial stroke. Using the Barthel Index, which is a clinical tool that assesses functional status, the study showed that AF patients suffering from a stroke had significantly lower scores than the non-AF patients in the acute period. This persisted at three- and six-month intervals in those that survived. Approximately 75% of AF patients were moderate to severely dependent in their activities of daily living (ADL) at the three-month mark [9]. In summary, elderly patients with AF are at an increased risk for stroke and appear to have worse outcomes when they do suffer a stroke.

Epidemiology and impact of falls in the elderly 

Falls can occur in the elderly in a variety of settings from hospitals to homes in the community. In general, adults residing in hospitals or long-term care facilities fall more frequently than those in the community. In a prospective study by Abreu et al. conducted in three hospitals in Brazil, falls were as frequent as 12.6 per 1,000 patients/day [10]. Patients with low educational levels, polypharmacy, visual impairment, gait impairment, or urinary incontinence were more prone to falls. Rubenstein and Josephson provided an overview of the epidemiology of falls in the elderly both in the community and institutions [12]. They reported that the incidence rates from multiple prospective studies were between 0.2 and 1.6 falls per individual per year with the incidence increasing with advancing age, individuals 80 years of age and older having the highest incidence. The overall incidence of falls experienced by elderly residing in institutions was also noted to be substantially higher than in the elderly living in the community. This difference was attributed to the condition of the individual in an institutionalized setting versus community as well as the more accurate reporting of falls within the institutions [11,12]. Tinetti et al. found that nearly 33% of adults 65 years and older in the community experience at a minimum one fall per year [4].

In addition, falls in the elderly, both in the community and inpatient settings, can lead to diminished quality of life. It has been found that in adults 65 years and older in a community setting, one-third fall at least one time per year and nearly 5% of falls will result in a fracture because of osteoporosis [4]. Individuals 80 years and older, based on self-reported data, have the highest incidence. This incidence is likely higher as individuals may underreport [11].

Are we using anticoagulation enough?

In a cross-sectional study of 682 hospitalized patients with AF or atrial flutter 80 years or older in Montreal, Lefebvre et al. examined the association between thromboembolic risk, bleeding risk, frailty, and the usage of anticoagulation [14]. Different clinical tools were used to assess each of the aforementioned including the Clinical Frailty Scale (CFS), which summarizes the general fitness or frailty of an elderly adult, CHADS2 score, and HAS-BLED score, which assesses the annual risk of major bleeding in patients with AF [13]. Nearly 70% of patients in this study received anticoagulation. Based on the scoring systems, those patients at high risk of stroke and/or absence of severe frailty were more likely to be on anticoagulation. Those with a high risk of bleeding and/or those who were severely frail were found not to be on anticoagulation. The study was uncertain if a 70% rate of anticoagulation is acceptable and advocate for further longitudinal investigations [14]. Although clinical tools are available to guide therapy, many physicians remain conflicting regarding anticoagulation in the elderly with AF.

Do the risks of falls really outweigh the benefits of anticoagulation?

Falls carry the risk of further injury including fractures, laceration, and even death. Physicians face a conundrum when making the decision to anticogulate an elderly patient with AF at high risk of stroke, but who also may be at risk for falls. Should one proceed with anticoagulation? Do the risks really outweigh the benefits?

Gage et al. examined the incidence of intracranial hemorrhage (ICH) in patients with AF at high risk for falls [15]. Approximately 33% of patients at high risk for falls were noted to be on warfarin. Even with the low utilization of warfarin, patients at high risk for falls had 2.8 ICHs per 100 patient-years, more than double the 1.1 rate of ICHs found in other patients. This increase in ICH was secondary to a higher incidence in ICH resulting from trauma, which was nearly fourfold that of other patients. The 30-day mortality following an ICH in patients on warfarin was 51.8% compared to those not on warfarin at 33.6%. Interestingly, the study concluded that anticoagulation should be used in patients with moderate to high risk of stroke despite having a high fall risk and its affiliation with ICH. This conclusion was derived from the fact that in patients at high risk for falls and a CHADS2 score of 2 or greater, warfarin was linked with a 25% relative risk reduction in out-of-hospital death, hospitalizations for stroke, myocardial infarction, and hemorrhage. As a caveat patient should be counselled on fall precautions prior to the initiation of warfarin [15].

A population-based study by Bjorck et al. found that warfarin was the most significant risk factor for ICH [16]. However, the risk for an ischemic stroke was nearly ninefold higher than the risk of an ICH in patients with AF regardless of anticoagulation status. They thus concluded that the benefits to warfarin treatment in the elderly far outweigh the risk of treatment.

Man-Son-Hing and Laupacis constructed a decision analysis which stated that the risk of falling in the elderly and the potential head trauma associated with it should not prevent these patients from receiving anticoagulation [17]. If patients were to be prescribed anticoagulation, the risk of developing a subdural hematoma (SDH) as a sequela of a fall is very low. A patient with AF needs to fall nearly 300 times in one year for the risk of an SDH to be greater than the benefit of preventing a stroke with anticoagulation therapy [17,18].

Is aspirin an alternative to anticoagulation for the prevention of stroke in patients with high risk of falls?

In the Birmingham Atrial Fibrillation Treatment of Aged Study (BAFTA), a randomized control trial of 973 patients, Mant et al. investigated the use of aspirin versus warfarin in patients with AF 75 years or older in a primary care setting [19]. Patients were either randomized to receive warfarin or aspirin. The study noted 21 episodes of either stroke, intracranial hemorrhage, or systemic emboli, in the group receiving warfarin compared to 48 episodes in the group receiving only aspirin. The frequency of major bleeds was essentially no different between the two groups. In patients in this age group, eligible and willing to receive anticoagulation, the data lend support in favor of the use of anticoagulation [19].

## Conclusions

AF is the most common arrythmia of clinical significance and the incidence increases as we get older. Although elder population are at an increased risk of falls, anticoagulation in this population is recommended as the benefit of stroke risk reduction outweighs the risks of intracranial bleeding in this population. Aspirin should not be used as an alternative to anticoagulation for older patients that are eligible for anticoagulation.
